# Supplementary material for: Can ultrasound parameters predict the diagnostic yield of diuretic renal scintigraphy in pediatric hydronephrosis?
Source: PLoS One. 2025 May 19;20(5):e0324446. doi: 10.1371/journal.pone.0324446 (PMC12088069; doi:10.1371/journal.pone.0324446)
Supplement: Attachment — Submitted filename: Response to Reviewers.docx [file pone.0324446.s003.docx]

**Response to Reviewers**

Dear Editor, PLOS ONE

Regarding the submission of the manuscript entitle “**Can ultrasound parameters predict the diagnostic yield of diuretic renal scintigraphy in pediatric hydronephrosis?**”, I extend my appreciation to the reviewers and the editorial office for reviewing and providing valuable comments to help improve the manuscript. I have revised the manuscript as per the recommendations, as follows. The revised texts are highlighted yellow in the revised manuscript.

| **Comments** | **Authors’ response** | **Revised text** |
| --- | --- | --- |
| 1. Please ensure that your manuscript meets PLOS ONE's style requirements, including those for file naming. | File names are renamed to comply with PLOS ONE's style requirements. | Fig1.tif  Fig2.tif  Fig3.tif  Fig4.tif  S1_Dataset.csv |
| 2. Please include captions for your Supporting Information files at the end of your manuscript, and update any in-text citations to match accordingly. | A caption for the Supporting Information file is included at the end of the revised manuscript. | **Supporting information**  **S1 Dataset**. Anonymized patient-level dataset |
| 3. Please review your reference list to ensure that it is complete and correct. If you have cited papers that have been retracted, please include the rationale for doing so in the manuscript text, or remove these references and replace them with relevant current references. Any changes to the reference list should be mentioned in the rebuttal letter that accompanies your revised manuscript. If you need to cite a retracted article, indicate the article’s retracted status in the References list and also include a citation and full reference for the retraction notice. | - The references were checked and revised to comply with the Vancouver style. - An additional reference is added as per recommendation from the reviewer (reference number 10) with the references that follow renumbered accordingly. | 10. Jacobson DL, Flink CC, Johnson EK, Maizels M, Yerkes EB, Lindgren BW, et al. The correlation between serial ultrasound and diuretic renography in children with severe unilateral hydronephrosis. J Urol. 2018 Aug;200(2):440–7. |
| Reviewer #2: |  |  |
| 1) Can some anotomical changes occur in the 6 month time gap between kidney US and DRS? What if the exclusion criteria is more tight (3 months or lower)? Will that make any difference in the results? | We agree that this is an important point, and it is one that we have considered when initiating the study. We believe that anatomical changes that could occur is possible, but should not be so pronounced as to invalidate the results of the study. This is because according to the “Pediatric congenital hydronephrosis (ureteropelvic junction  obstruction): Medical management guide” (reference 5), children with hydronephrosis are initially managed medically with serial ultrasound follow-up every 3-6 months, which suggests that significant changes in the kidney anatomy would not occur in a short time frame.  However, to explore if including only those that underwent DRS and US within 3 months (90 days) of each other, we did a subgroup analysis to find the ROCs of US parameters in 44 patients whose imaging tests were done within a 3-month window. The results are presented in a new table (Table 6). The AUCs are still poor, so we believe that the time window did not have a significant effect on the outcome and the key message of this study. | The median time window between DRS and US was 44 days. To further examine if the time window between DRS and US could have affected the predictiveness of US parameters, a subgroup analysis was done in 44 of 67 patients (65.7%) who underwent both modalities within ≤ 90 days of each other. The results are presented in table 6. The AUCs of the US parameters did not improve, ranging from 0.46 – 0.56, with all having 95% confidence intervals crossing 0.5. This suggests that the time window is not an effect modifier on the predictiveness of US parameters.   |
| 2) are there any previous studies that have similarly explored the predictive ability of Kidney US? The authors have not mentioed about them. It would be intersting to know what is the outcome in the previous studies. | There have been several studies which explored the predictiveness of US on predicting the “obstruction pattern” on DRS (reference numbers 11 - 15). However, there have not been studies that explored the predictiveness of US in predicting “non-diagnostic” DRS. We did additional searching and the closest we could find is a study by Jacobson et al. (reference 10) which analyzed the correlation between US and DRS, but did not analyze the ROC. We added this reference and added this discussion point. | A previous study by Jacobson et al. found a poor correlation between DRS and US findings in children with hydronephrosis with a Spearman correlation coefficient of only 0.24 [10]. However, to our knowledge, this current study is the first to examine the predictiveness of US parameters in relation to diagnostic vs. non-diagnostic DRS results. |
| 3) Authors can mention any limiations in the current study. Also, mention the future directions for the researchers reading the manuscript. | We added a paragraph about the limitations and future directions at the end of the discussion section. | The main limitation of this study is its retrospective nature. Although all US studies were done by pediatric radiologists, since US is a highly operator-dependent modality, variations at the time the US studies were done are difficult to avoid. To address this issue, in this study, all US parameters were remeasured by one experienced pediatric radiologist (N.T.). Future prospective studies should aim to integrate multiple clinical, functional, and anatomical parameters to enhance prediction accuracy and improve patient selection for DRS. |
| While revising your submission, please upload your figure files to the Preflight Analysis and Conversion Engine (PACE) digital diagnostic tool | I uploaded the figure file to PACE as instructed, and downloaded the PACE-adjusted version, and reuploaded them to the Editorial Manager submission system. | Fig1.tif  Fig2.tif  Fig3.tif  Fig4.tif |
